# A machine learning model for classifying G-protein-coupled receptors as agonists or antagonists

**DOI:** 10.1186/s12859-022-04877-7

**Published:** 2022-08-18

**Authors:** Jooseong Oh, Hyi-thaek Ceong, Dokyun Na, Chungoo Park

**Affiliations:** 1 School of Biological Sciences and Technology, Chonnam National University, Gwangju, 61186 Republic of Korea; 2 Department of Multimedia, Chonnam National University, Yeosu, 59626 Republic of Korea; 3 Department of Biomedical Engineering, Chung-Ang University, Seoul, 06974 Republic of Korea

**Keywords:** G-protein-coupled receptors, GPCR–ligand interactions, GPCR agonists and antagonists, Machine learning, Two-step random forest classification

## Abstract

**Background:**

G-protein coupled receptors (GPCRs) sense and transmit extracellular signals into the intracellular machinery by regulating G proteins. GPCR malfunctions are associated with a variety of signaling-related diseases, including cancer and diabetes; at least a third of the marketed drugs target GPCRs. Thus, characterization of their signaling and regulatory mechanisms is crucial for the development of effective drugs.

**Results:**

In this study, we developed a machine learning model to identify GPCR agonists and antagonists. We designed two-step prediction models: the first model identified the ligands binding to GPCRs and the second model classified the ligands as agonists or antagonists. Using 990 selected subset features from 5270 molecular descriptors calculated from 4590 ligands deposited in two drug databases, our model classified non-ligands, agonists, and antagonists of GPCRs, and achieved an area under the ROC curve (AUC) of 0.795, sensitivity of 0.716, specificity of 0.744, and accuracy of 0.733. In addition, we verified that 70% (44 out of 63) of FDA-approved GPCR-targeting drugs were correctly classified into their respective groups.

**Conclusions:**

Studies of ligand–GPCR interaction recognition are important for the characterization of drug action mechanisms. Our GPCR–ligand interaction prediction model can be employed in the pharmaceutical sciences for the efficient virtual screening of putative GPCR-binding agonists and antagonists.

**Supplementary Information:**

The online version contains supplementary material available at 10.1186/s12859-022-04877-7.

## Background

G-protein coupled receptors (GPCRs) belong to membrane protein families that sense and transmit extracellular signals to the intracellular region by regulating G proteins. GPCRs are involved in diverse signaling pathways triggered by hormones and neurotransmitters, and participate in cell growth, differentiation, vision, olfaction, and gustatory system [1]. When a ligand binds to a GPCR, the receptor undergoes a conformational change that can either activate (called an agonist) or inhibit (called an antagonist) signal transduction pathways [2]. Approximately one-third of the drugs on the market target GPCRs [2, 3] and are used to treat various human diseases including cardiac malfunction, asthma, and migraines [4]. In 2017, Hauser et al. reported that approximately 34% (475 drugs) of all US FDA (Food and Drug Administration)-approved drugs act on GPCR targets, and that most agents in clinical trials target novel GPCRs [5].

Owing to recent technological advances in receptor pharmacology, new avenues for GPCR drug discovery have emerged that diverge from the traditional view of signal transduction as a linear chain of events involving the heterotrimeric G proteins. However, GPCR drug discovery has long been focused on the identification of new compounds targeting GPCRs and their ligand binding sites. The classification of the agonist and antagonist properties of existing and newly discovered ligands is needed to optimize drug efficacy and develop appropriate therapeutic strategies that selectively activate or block relevant pathways.

Using a support vector machine (SVM) learning algorithm with 4884 chemical descriptors as input, Bushdid et al. [6] virtually screened 258 chemical compounds and determined agonists for the human G-protein-coupled odorant receptor (OR) 51E1 as well as human receptors OR1A1 and OR2W1, and mouse receptor MOR256-3. The predicted novel agonists were identified with a hit rate of 39–50%. Two newly identified agonists for OR51E1 were functionally validated through in vitro assays. In addition, to predict ligands and their roles in the human olfactory receptor OR1G1, Jabeen and Ranganathan [7] built classification models (SVM, random forest, naïve bayes, and neural networks) based on 13 relevant features for a dataset of 74 agonists and 74 antagonists. The area under the ROC curve (AUC) was 0.652–0.827. Using over 200,000 compounds, the best performing classifier, naïve bayes model, predicted 37 compounds as agonists for OR1G1 with > 80% probability score.

In this study, we developed a ligand-based machine learning model to identify novel human GPCR agonists and antagonists, irrespective of GPCR types. Using the existing knowledge-base to predict ligand activity according to similarities/dissimilarities of known active ligands, we designed two-step machine learning models that first identify the ligands binding to GPCRs and then classify the ligands as agonists or antagonists. GPCR ligand information from the International Union of Basic and Clinical Pharmacology (IUPHAR)/British Pharmacological Society (BPS) Guide to PHARMACOLOGY database (GtoPdb) [8] and Context-Oriented Directed Associations (CODA) [9] database were used to train two random forest (RF) models that will act independently but successively to classify query components into non-ligands, agonists, and antagonists of GPCRs. The optimal performance parameters for the integrated two-step models were AUC = 0.795, accuracy = 0.733, sensitivity = 0.716, and specificity = 0.744. Hence, our model allowed us to understand the molecular mechanisms of GPCR–ligand interactions. This model can be employed in the pharmaceutical sciences to screen novel drugs and therapeutic agents.

## Results and discussion

### Data collection and preprocessing

Out of 14,659 initially available human ligand-target interactions, 4590 ligand-target pairs were analyzed. We obtained 1058 and 1150 ligands that act as agonists (hereafter called GPCR-agonist) and antagonists (hereafter called GPCR-antagonist), respectively; the remaining 2382 ligands were classified as non-ligands of GPCRs (hereafter called GPCR-nontarget).

To eliminate potentially redundant ligands, ligands were clustered with their ECFP4 (extended connectivity fingerprints of bond diameter 4) fingerprints encoding the ligand’s structural characteristics as a vector [10] using an agglomerative hierarchical clustering method. This algorithm iteratively merges subclusters based on their similarity (above 0.8 in this study [11]) considering interconnectivity and closeness of the clusters [12]. Only representative ligands in each cluster were used for training and test dataset. Consequently, 758 GPCR-agonists, 950 GPCR-antagonists, and 2206 GPCR-nontargets were selected for further analysis.

### Molecular descriptor calculation and feature selection

**Fig. 1:**
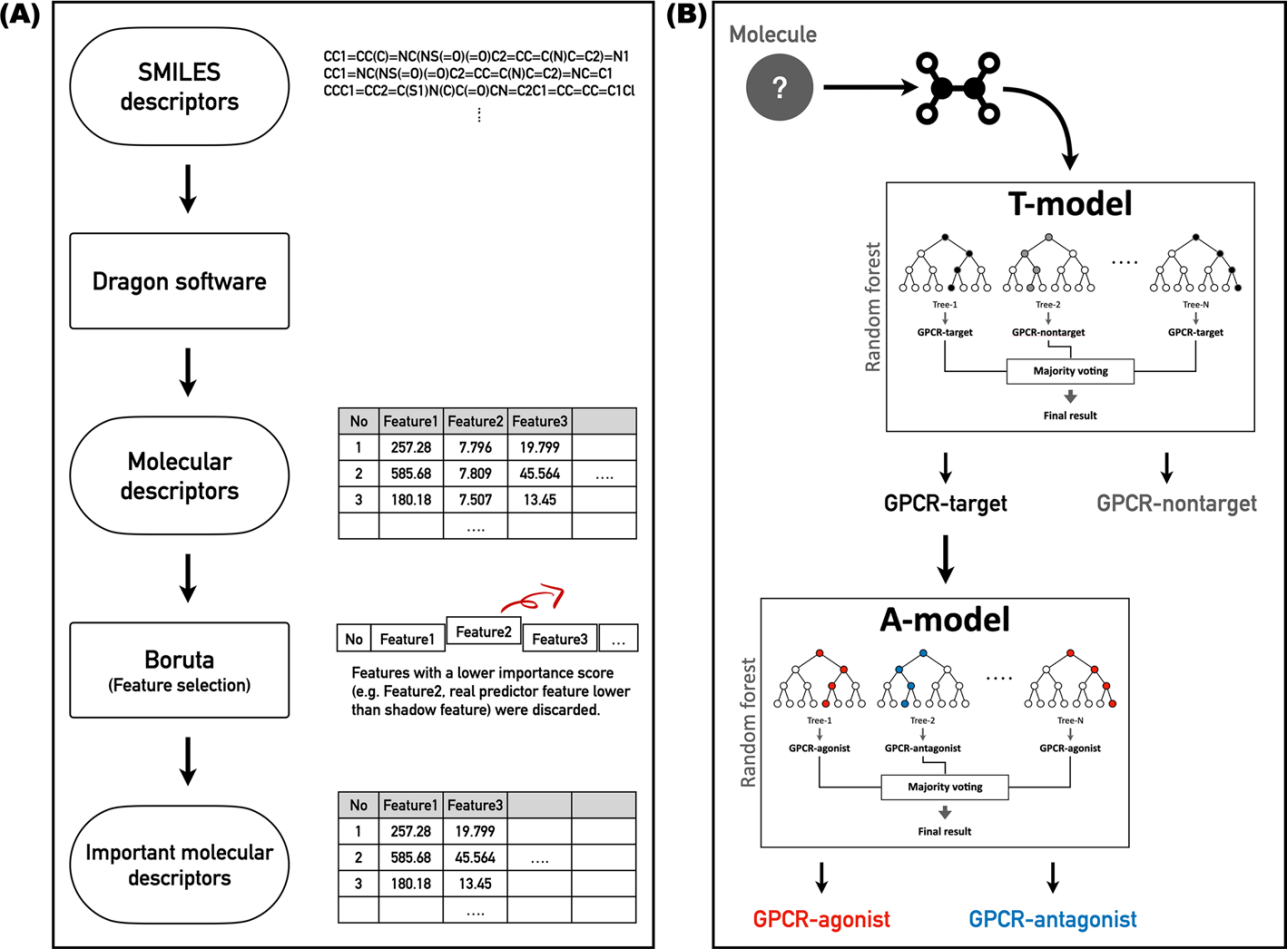
Overall workflows of **A** feature selection process and **B** two-step binary-class RF models

We calculated 5270 molecular descriptors using Dragon software, and they were used for feature selection. Using *Boruta* algorithm that performs the comparison of the real predictor features with those of random (so-called shadow) variables, 990 selected predictor features (Additional file 1) with significantly larger importance values were taken as inputs for machine learning classifiers (Fig. 1A).

### Machine learning model construction and evaluation

We designed two-step binary-class classifiers, given their superior accuracy estimates compared to multi-class classifiers [13]. The first model (T-model) predicted GPCR-target or GPCR-nontarget; the second model (A-model) predicted GPCR-agonist or GPCR-antagonist (Fig. 1B). Specifically, when a query molecule is input, the T-model predicts whether or not the molecule is a GPCR ligand. If not, it is classified as a GPCR-nontarget molecule. If classified as a GPCR-target, the A-model predicts whether it acts as a GPCR agonist or antagonist.

For the T-model, 1708 GPCR-target (from 758 GPCR-agonists and 950 GPCR-antagonists) and 2206 GPCR-nontarget were used in the training dataset. Because no statistical model functions at 100% accuracy, some of the GPCR-nontarget classified molecules could potentially interact with GPCRs; thus, we used all of the available data to minimize the data imbalance [14, 15]. For the A-model, we used 758 GPCR-agonists and 950 GPCR-antagonists in the training data set.

The T-model and A-model were built separately using the RF classifier and were evaluated using the leave-one-out cross-validation (LOO-CV) method. The T-model and A-model achieved an AUC of 0.787 and 0.823, respectively. The final integrated two-step model produced an AUC of 0.795 (accuracy = 0.733, sensitivity = 0.716, and specificity = 0.744) (Table 1).

**Table 1:** Performance parameters of the two-step binary-class models

Model	Accuracy	Sensitivity	Specificity	PPV^1^	NPV^2^	F1^3^	MCC^4^	AUC^5^
T-model	0.726	0.699	0.744	0.652	0.783	0.675	0.439	0.787
A-model	0.758	0.780	0.744	0.647	0.849	0.707	0.510	0.823
Integrated^6^	0.733	0.716	0.744	0.651	0.797	0.682	0.454	0.795

^1^Positive predictive value

^2^Negative predictive value

^3^Harmonic means of PPV and sensitivity

^4^Matthews correlation coefficient

^5^Area under the ROC curve

^6^The performance values were measured with micro-average

### Model validation with FDA-approved GPCR drugs

To validate our model under different experimental conditions, we used FDA-approved GPCR-targeting drugs. Data for 134 drugs were collected, of which data for 63 drugs with ligand-binding types and SMILES (simplified molecular input line entry system) descriptors were used for the model validation procedures.

Our T-model predicted that 52 of 63 (82.5%) drugs could interact with GPCRs. According to the A-model, 44 of the 52 GPCR-target drugs (84.6%) were correctly categorized as agonists or antagonists. Consequently, 44 out of 63 (69.8%) FDA-approved GPCR-targeting drugs were correctly classified into their respective groups (GPCR-agonist, GPCR-antagonist, GPCR-nontarget) (Table 2). In addition to the positive data, our T-model was also tested on negative dataset. To this end, we collected 1278 GPCR-nontarget drugs (out of 14,594 drugs) from DrugBank database. After excluding ligands for which descriptors were not calculated by Dragon software, we retained 982 drugs as GPCR-nontarget drugs. 808 of 982 GPCR-nontarget drugs (82.3%) were correctly predicted by our T-model (Additional file 2). Though our study considered a relatively small sample size, our results clearly showed that the integrated two-step RF models had a high and balanced prediction accuracy. Future studies should consider the practical compatibility of virtual screening with larger sample size datasets and more complex models associated with signaling pathways.

**Table 2:** Model evaluation using FDA-approved GPCR drugs

Drug name	Target	FDA-approved action	T-model-predicted action	A-model-predicted action	References
Beclometasone dipropionate	Glucocorticoid receptor	GPCR-agonist	GPCR-target	GPCR-agonist	[16]
Adenosine	Adenosine receptor A1	GPCR-agonist	**GPCR-nontarget**	GPCR-agonist	[16]
Regadenoson	Adenosine receptor A2a	GPCR-agonist	GPCR-target	GPCR-agonist	[16]
Nicardipine	Alpha-1A adrenergic receptor	GPCR-antagonist	GPCR-target	GPCR-antagonist	[16]
Oxymetazoline	Alpha-2B adrenergic receptor	GPCR-agonist	GPCR-target	GPCR-agonist	[16]
Prazosin	Alpha-1A adrenergic receptor	GPCR-antagonist	GPCR-target	GPCR-antagonist	[16]
Apraclonidine	Alpha-2A adrenergic receptor	GPCR-agonist	GPCR-target	GPCR-agonist	[16]
Dexmedetomidine	Alpha-2A adrenergic receptor	GPCR-agonist	GPCR-target	GPCR-agonist	[16]
Acebutolol	Beta-1 adrenergic receptor	GPCR-agonist	**GPCR-nontarget**	**GPCR-antagonist**	[16]
Mirabegron	Beta-3 adrenergic receptor	GPCR-agonist	GPCR-target	GPCR-agonist	[16]
Candesartan	Type-1 angiotensin II receptor	GPCR-antagonist	GPCR-target	GPCR-antagonist	[16]
Pentagastrin	Gastrin/cholecystokinin type B receptor	GPCR-agonist	**GPCR-nontarget**	GPCR-agonist	[16]
Maraviroc	C–C chemokine receptor type 5	GPCR-antagonist	GPCR-target	GPCR-antagonist	[16]
Biperiden	Muscarinic acetylcholine receptor M1	GPCR-antagonist	GPCR-target	GPCR-antagonist	[16]
Propantheline	Muscarinic acetylcholine receptor M1	GPCR-antagonist	GPCR-target	GPCR-antagonist	[16]
Umeclidinium	Muscarinic acetylcholine receptor M1	GPCR-antagonist	GPCR-target	GPCR-antagonist	[16]
Nabilone	Cannabinoid receptor 2	GPCR-agonist	GPCR-target	GPCR-agonist	[16]
Zafirlukast	Cysteinyl leukotriene receptor 1	GPCR-antagonist	GPCR-target	GPCR-antagonist	[16]
Dopamine	Dopamine D2 receptor	GPCR-agonist	GPCR-target	GPCR-agonist	[16]
Ambrisentan	Endothelin-1 receptor	GPCR-antagonist	GPCR-target	GPCR-antagonist	[16]
Bosentan	Endothelin-1 receptor	GPCR-antagonist	GPCR-target	GPCR-antagonist	[16]
Vorapaxar	Proteinase-activated receptor 1	GPCR-antagonist	GPCR-target	GPCR-antagonist	[16]
Baclofen	Gamma-aminobutyric acid type B receptor subunit 2	GPCR-agonist	**GPCR-nontarget**	GPCR-agonist	[16]
Estradiol	Estrogen receptor alpha	GPCR-agonist	**GPCR-nontarget**	GPCR-agonist	[16]
Levodopa	Dopamine D1 receptor	GPCR-agonist	GPCR-target	GPCR-agonist	[16]
Dronabinol	Cannabinoid receptor 1	GPCR-agonist	**GPCR-nontarget**	GPCR-agonist	[16]
Bumetanide	Solute carrier family 12 member 1	GPCR-antagonist	GPCR-target	**GPCR-agonist**	[16]
Nicotinic acid	Hydroxycarboxylic acid receptor 3	GPCR-agonist	GPCR-target	GPCR-agonist	[16]
Suvorexant	Orexin receptor type 1	GPCR-antagonist	GPCR-target	GPCR-antagonist	[16]
Cetirizine	Histamine H1 receptor	GPCR-antagonist	GPCR-target	**GPCR-agonist**	[16]
Betazole	Histamine H2 receptor	GPCR-agonist	GPCR-target	GPCR-agonist	[16]
Clozapine	Dopamine D2 receptor	GPCR-antagonist	GPCR-target	GPCR-antagonist	[16]
Frovatriptan	5-hydroxytryptamine receptor 1D	GPCR-agonist	GPCR-target	GPCR-agonist	[16]
Eletriptan	5-hydroxytryptamine receptor 1D	GPCR-agonist	GPCR-target	GPCR-agonist	[16]
Ergotamine	5-hydroxytryptamine receptor 1D	GPCR-agonist	GPCR-target	**GPCR-antagonist**	[16]
Amoxapine	Sodium-dependent serotonin transporter	GPCR-antagonist	**GPCR-nontarget**	GPCR-antagonist	[16]
Lurasidone	Dopamine D2 receptor	GPCR-antagonist	GPCR-target	GPCR-antagonist	[16]
Chloroquine	Glutathione S-transferase A2	GPCR-antagonist	GPCR-target	**GPCR-agonist**	[16]
Tasimelteon	Melatonin receptor type 1A	GPCR-agonist	GPCR-target	GPCR-agonist	[16]
Niclosamide	DNA	GPCR-antagonist	**GPCR-nontarget**	GPCR-antagonist	[16]
Levocabastine	Histamine H1 receptor	GPCR-antagonist	GPCR-target	**GPCR-agonist**	[16]
Naltrexone	Delta-type opioid receptor	GPCR-antagonist	GPCR-target	GPCR-antagonist	[16]
Anileridine	Mu-type opioid receptor	GPCR-agonist	GPCR-target	**GPCR-antagonist**	[16]
Alfentanil	Mu-type opioid receptor	GPCR-agonist	GPCR-target	**GPCR-antagonist**	[16]
Cangrelor	P2Y purinoceptor 12	GPCR-antagonist	GPCR-target	GPCR-antagonist	[16]
Treprostinil	Prostacyclin receptor	GPCR-agonist	GPCR-target	GPCR-agonist	[16]
Indomethacin	Prostaglandin G/H synthase 2	GPCR-antagonist	GPCR-target	**GPCR-agonist**	[16]
Prostaglandin E1	Prostaglandin E2 receptor EP2 subtype	GPCR-agonist	GPCR-target	GPCR-agonist	[16]
Prostaglandin E2	Prostaglandin E2 receptor EP2 subtype	GPCR-agonist	**GPCR-nontarget**	GPCR-agonist	[16]
Misoprostol	Prostaglandin E2 receptor EP3 subtype	GPCR-agonist	GPCR-target	GPCR-agonist	[16]
Latanoprost	Prostaglandin F2-alpha receptor	GPCR-agonist	GPCR-target	GPCR-agonist	[16]
Epoprostenol	P2Y purinoceptor 12	GPCR-agonist	GPCR-target	GPCR-agonist	[16]
Sonidegib	Smoothened homolog	GPCR-antagonist	GPCR-target	GPCR-antagonist	[16]
Aprepitant	Neurokinin 1 receptor	GPCR-antagonist	GPCR-target	GPCR-antagonist	[16]
Iloprost	Prostacyclin receptor	GPCR-agonist	GPCR-target	GPCR-agonist	[16]
Droxidopa	Alpha-1A adrenergic receptor	GPCR-agonist	GPCR-target	GPCR-agonist	[5]
Naloxegol	Mu-type opioid receptor	GPCR-antagonist	**GPCR-nontarget**	**GPCR-agonist**	[5]
Netupitant	Neurokinin 1 receptor	GPCR-antagonist	GPCR-target	GPCR-antagonist	[5]
Olodaterol	Beta-2 adrenergic receptor	GPCR-agonist	GPCR-target	GPCR-agonist	[5]
Rolapitant	Neurokinin 1 receptor	GPCR-antagonist	GPCR-target	GPCR-antagonist	[5]
Selexipag	Prostacyclin receptor	GPCR-agonist	GPCR-target	GPCR-agonist	[5]
Pimavanserin	5-hydroxytryptamine receptor 2A	GPCR-agonist	GPCR-target	GPCR-agonist	[5]
Naldemedine	Mu-type opioid receptor	GPCR-antagonist	**GPCR-nontarget**	GPCR-antagonist	[5]

Note that incorrectly predicted events are shown in bold

## Conclusion

Because the GPCRs are involved in diverse cellular signaling transductions and therefore play essential and important roles in pharmaceutical research, they have long been considered as prime targets for drug discovery. However, unlike other cellular proteins, experimental screening of GPCR structure–function and ligand-identification is expensive and time-consuming. Machine learning-based approaches have recently gained popularity in GPCR-based virtual drug discovery. In this study, we developed in-silico models to predict GPCR-agonists and GPCR-antagonists with reasonably high accuracy. The key contribution of this work is two folds: first one is presenting a GPCR-type independent classification model that could classify both GPCR agonists and antagonists together, regardless of the GPCR types, and second is using over 14,000 of publicly available ligand-target interaction data that could make the model more accurate and could be used in future similar studies. Although our prediction models require further testing, they could be applied in drug discovery technologies to predict putative GPCR-binding ligands from millions of unlabeled chemical compounds.

## Methods

### Data acquisition

We acquired pharmacological datasets relating to ligand-activity-target relationships from the GtoPdb (https://www.guidetopharmacology.org) [8], including data for over 1700 drug targets with over 9000 related ligands, and the CODA network database [9], including drug–drug target associations with related molecular, phenomic, and anatomical variables. Out of 14,659 human ligand-target interactions, 4590 ligand-target pairs were analyzed in this study and included both a ligand-binding type (e.g., agonist and antagonist) and a SMILES descriptor.

We collated a list of the FDA-approved GPCR-targeting drugs [5, 16] and screened the DrugBank database [17] for ligand-binding types and SMILES descriptors related to these drugs.

ECFP4 fingerprints were calculated using Dragon software (version 7.0.10) [18], and the Tanimoto index [19] was used to determine the similarity between ligands.

### Feature selection

Dragon software (version 7.0.10) [18] was used to calculate the chemical and physical properties (molecular descriptors) of chemicals from their SMILES as an input. These chemoinformatic properties include 1D descriptors, such as the number of atom types and structural fragments of the molecule, and 2D descriptors, such as structural features, logP, and connectivity indices [18, 20].

We applied the *Boruta* packages (version 7.0.0) [21] with default parameters to obtain the best subset of descriptors. To screen the key features in each class, the FSelector package [22] in R software was used.

### Machine learning model and performance evaluation

We applied a RF machine learning model, using the randomForest function in the R randomForest package [23]. For the main two parameters, the number of random explanatory variables for splitting each tree node, *mtry*, and the number of trees, *ntree*, were set at number of features and 100, respectively.

To validate the constructed RF model, we used the LOO-CV for method selection [24]. To obtain the performance measurement values (true positive, TP; true negative, TN; false positive, FP; false negative, FN) of the integrated two-step models, a micro-average calculation [25] was used.

## Supplementary Information

**Additional file 1**: Selected 990 features after applying Boruta algorithm.

**Additional file 2**: Model evaluation using GPCR-nontarget drugs from DrugBank and UniProt database.

## Abbreviations

AUC: Area under the ROC curve
BPS: British pharmacological society
CODA: Context-oriented directed associations
ECFP4: Extended connectivity fingerprints of bond diameter 4
FDA: Food and drug administration
GPCRs: G-protein coupled receptors
GtoPdb: Guide to PHARMACOLOGY database
IUPHAR: The International Union of basic and clinical pharmacology
LOO-CV: Leave-one-out cross-validation
RF: Random forest
SMILES: Simplified molecular input line entry system
